# 1,4-Dibromo-2,5-dimeth­oxy­benzene

**DOI:** 10.1107/S1600536810023548

**Published:** 2010-06-26

**Authors:** Zhong-Hua Luo, Jin Chang, Mei-Li Feng, Qin Zhang, Hong-Jun Zhu

**Affiliations:** a Department of Applied Chemistry, College of Science, Nanjing University of Technology, Nanjing 210009, People’s Republic of China

## Abstract

The asymmetric unit of the title compound, C_8_H_8_Br_2_O_2_, contains one half-mol­ecule, the complete mol­ecule being generated by inversion symmetry.

## Related literature

For standard bond lengths, see: Allen *et al.* (1987 ▶). For the synthetic procedure, see: Lopez-Alvarado *et al.* (2002 ▶). For potential uses of compounds derived from the title compound, see: Chen *et al.* (2006 ▶).
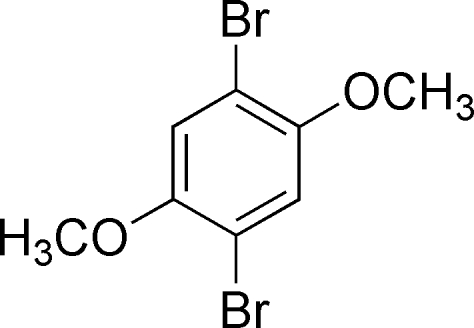


## Experimental

### 

#### Crystal data

C_8_H_8_Br_2_O_2_
*M* *_r_* = 295.94
Monoclinic, 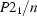

*a* = 6.573 (1) Å
*b* = 8.438 (2) Å
*c* = 8.756 (2) Å
β = 90.14 (3)°
*V* = 485.6 (2) Å^3^
*Z* = 2
Mo *K*α radiation
μ = 8.30 mm^−1^
*T* = 298 K
0.20 × 0.10 × 0.10 mm

#### Data collection

Enraf–Nonius CAD-4 diffractometer
Absorption correction: ψ scan (North *et al.*, 1968 ▶) *T*_min_ = 0.288, *T*_max_ = 0.491
1761 measured reflections
884 independent reflections
622 reflections with *I* > 2σ(*I*)
*R*_int_ = 0.112
3 standard reflections every 200 reflections  intensity decay: 1%

#### Refinement

*R*[*F* ^2^ > 2σ(*F* ^2^)] = 0.044
*wR*(*F* ^2^) = 0.102
*S* = 1.01
884 reflections
55 parameters
H-atom parameters constrained
Δρ_max_ = 0.58 e Å^−3^
Δρ_min_ = −0.41 e Å^−3^

### 

Data collection: *CAD-4 Software* (Enraf–Nonius, 1985 ▶); cell refinement: *CAD-4 Software*; data reduction: *XCAD4* (Harms & Wocadlo, 1995 ▶); program(s) used to solve structure: *SHELXS97* (Sheldrick, 2008 ▶); program(s) used to refine structure: *SHELXL97* (Sheldrick, 2008 ▶); molecular graphics: *SHELXTL* (Sheldrick, 2008 ▶); software used to prepare material for publication: *SHELXTL*.

## Supplementary Material

Crystal structure: contains datablocks I, lzh. DOI: 10.1107/S1600536810023548/im2205sup1.cif

Structure factors: contains datablocks I. DOI: 10.1107/S1600536810023548/im2205Isup2.hkl

Additional supplementary materials:  crystallographic information; 3D view; checkCIF report

## Figures and Tables

**Fig. 1.**
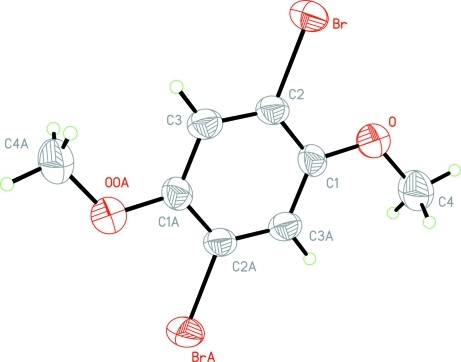
Molecular structure of the title compound showing the atom-numbering scheme. Displacement ellipsoids are drawn at the 40% probability level.

**Fig. 2.**
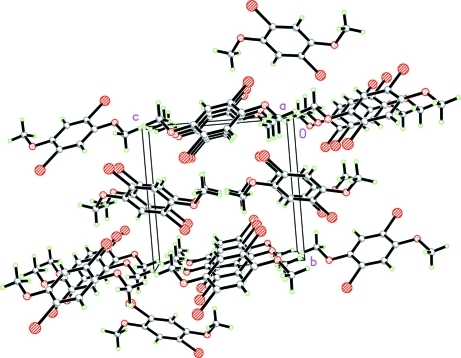
Molecular packing of the title compound.

## supplementary crystallographic information

### Comment

The title compound, 1,4-dibromo-2,5-dimethoxybenzene is an important intermediate in the synthesis of 4-(2',5'-dimethoxy-4'-acetylthiophenyl)phenyl-nonafluorobiphenyl, which can be used as molecular switch, transistor and in the manufacture of memory devices (Chen *et al.*, 2006). We report here the crystal structure of the title compound, (I).

The molecular structure of (I) is shown in Fig. 1. Bond lengths and angles are within normal ranges (Allen *et al.*, 1987).

The benzene ring is planar and it's center respresents a crystallographic center of inversion. So only half of the molecule was observed in the asymmetric unit. No hydrogen bond interactions were observed in the crystal structure.

### Experimental

The title compound, (I) was synthesized according to a literature method reported before (Lopez-Alvarado *et al.*, 2002). Single crystals were obtained by slow evaporation of a methanolic (25 ml) solution of the compound (0.30 g, 1.0 mmol) at room temperature for about 15 d.

### Refinement

H atoms were positioned geometrically, with C—H = 0.93 Å for aromatic H and 0.96 Å for methyl H, and constrained to ride on their parent atoms, with *U*_iso_(H) = *xU*_eq_(C/O), where *x* = 1.2 for aromatic H and *x* = 1.5 for other H.

### Crystal data

**Table:**

C_8_H_8_Br_2_O_2_	*F*(000) = 284
*M**_r_* = 295.94	*D*_x_ = 2.024 Mg m^−^^3^
Monoclinic, *P*2_1_/*n*	Mo *K*α radiation, λ = 0.71073 Å
Hall symbol: -P 2yn	Cell parameters from 25 reflections
*a* = 6.573 (1) Å	θ = 9–13°
*b* = 8.438 (2) Å	µ = 8.30 mm^−^^1^
*c* = 8.756 (2) Å	*T* = 298 K
β = 90.14 (3)°	Block, colourless
*V* = 485.6 (2) Å^3^	0.20 × 0.10 × 0.10 mm
*Z* = 2	

### Data collection

**Table:**

Enraf–Nonius CAD-4 diffractometer	622 reflections with *I* > 2σ(*I*)
Radiation source: fine-focus sealed tube	*R*_int_ = 0.112
graphite	θ_max_ = 25.3°, θ_min_ = 3.4°
ω/2θ scans	*h* = −7→7
Absorption correction: ψ scan (North *et al.*, 1968)	*k* = −10→0
*T*_min_ = 0.288, *T*_max_ = 0.491	*l* = −10→10
1761 measured reflections	3 standard reflections every 200 reflections
884 independent reflections	intensity decay: 1%

### Refinement

**Table:**

Refinement on *F*^2^	Primary atom site location: structure-invariant direct methods
Least-squares matrix: full	Secondary atom site location: difference Fourier map
*R*[*F*^2^ > 2σ(*F*^2^)] = 0.044	Hydrogen site location: inferred from neighbouring sites
*wR*(*F*^2^) = 0.102	H-atom parameters constrained
*S* = 1.01	*w* = 1/[σ^2^(*F*_o_^2^) + (0.03*P*)^2^ + 0.0*P*] where *P* = (*F*_o_^2^ + 2*F*_c_^2^)/3
884 reflections	(Δ/σ)_max_ < 0.001
55 parameters	Δρ_max_ = 0.58 e Å^−^^3^
0 restraints	Δρ_min_ = −0.41 e Å^−^^3^

### Special details

**Table:**

Geometry. All e.s.d.'s (except the e.s.d. in the dihedral angle between two l.s. planes) are estimated using the full covariance matrix. The cell e.s.d.'s are taken into account individually in the estimation of e.s.d.'s in distances, angles and torsion angles; correlations between e.s.d.'s in cell parameters are only used when they are defined by crystal symmetry. An approximate (isotropic) treatment of cell e.s.d.'s is used for estimating e.s.d.'s involving l.s. planes.
Refinement. Refinement of *F*^2^ against ALL reflections. The weighted *R*-factor *wR* and goodness of fit *S* are based on *F*^2^, conventional *R*-factors *R* are based on *F*, with *F* set to zero for negative *F*^2^. The threshold expression of *F*^2^ > σ(*F*^2^) is used only for calculating *R*-factors(gt) *etc*. and is not relevant to the choice of reflections for refinement. *R*-factors based on *F*^2^ are statistically about twice as large as those based on *F*, and *R*- factors based on ALL data will be even larger.

### Fractional atomic coordinates and isotropic or equivalent isotropic displacement parameters (Å^2^)

**Table:**

	*x*	*y*	*z*	*U*_iso_*/*U*_eq_	
Br	0.26386 (11)	0.73471 (7)	0.78061 (8)	0.0598 (3)	
O	−0.1091 (7)	0.5578 (5)	0.7000 (5)	0.0551 (11)	
C1	−0.0605 (9)	0.5256 (6)	0.8479 (6)	0.0394 (13)	
C2	0.1090 (9)	0.5986 (5)	0.9077 (7)	0.0400 (13)	
C3	0.1721 (9)	0.5752 (5)	1.0558 (7)	0.0433 (14)	
H3A	0.2881	0.6260	1.0922	0.052*	
C4	−0.2593 (11)	0.4649 (8)	0.6294 (8)	0.0649 (19)	
H4A	−0.2775	0.4993	0.5257	0.097*	
H4B	−0.3852	0.4761	0.6837	0.097*	
H4C	−0.2181	0.3558	0.6304	0.097*	

### Atomic displacement parameters (Å^2^)

**Table:**

	*U*^11^	*U*^22^	*U*^33^	*U*^12^	*U*^13^	*U*^23^
Br	0.0762 (5)	0.0516 (4)	0.0517 (5)	−0.0207 (3)	0.0162 (3)	0.0026 (3)
O	0.065 (3)	0.055 (2)	0.045 (3)	−0.011 (2)	−0.004 (2)	0.0031 (19)
C1	0.049 (3)	0.036 (3)	0.033 (3)	0.002 (3)	0.007 (3)	−0.003 (2)
C2	0.047 (3)	0.031 (3)	0.042 (4)	−0.004 (3)	0.009 (3)	−0.002 (2)
C3	0.049 (3)	0.034 (3)	0.046 (4)	−0.006 (3)	0.007 (3)	−0.003 (2)
C4	0.070 (5)	0.078 (4)	0.047 (5)	−0.004 (4)	−0.014 (4)	0.009 (4)

### Geometric parameters (Å, °)

**Table:**

Br—C2	1.897 (5)	C3—C1^i^	1.405 (7)
O—C1	1.361 (7)	C3—H3A	0.9300
O—C4	1.403 (8)	C4—H4A	0.9600
C1—C2	1.375 (8)	C4—H4B	0.9600
C1—C3^i^	1.405 (7)	C4—H4C	0.9600
C2—C3	1.375 (8)		
			
C1—O—C4	118.1 (5)	C2—C3—H3A	120.1
O—C1—C2	117.4 (5)	C1^i^—C3—H3A	120.1
O—C1—C3^i^	124.8 (5)	O—C4—H4A	109.5
C2—C1—C3^i^	117.8 (5)	O—C4—H4B	109.5
C1—C2—C3	122.5 (5)	H4A—C4—H4B	109.5
C1—C2—Br	118.9 (4)	O—C4—H4C	109.5
C3—C2—Br	118.6 (4)	H4A—C4—H4C	109.5
C2—C3—C1^i^	119.7 (5)	H4B—C4—H4C	109.5
			
C4—O—C1—C2	169.3 (5)	O—C1—C2—Br	−1.0 (6)
C4—O—C1—C3^i^	−11.4 (8)	C3^i^—C1—C2—Br	179.7 (4)
O—C1—C2—C3	179.8 (5)	C1—C2—C3—C1^i^	−0.5 (8)
C3^i^—C1—C2—C3	0.5 (8)	Br—C2—C3—C1^i^	−179.7 (4)

Symmetry codes: (i) −*x*, −*y*+1, −*z*+2.
